# Molecular characterization of *Treponema pallidum*-related rabbit-derived material following experimental inoculation with human chancre-derived clinical specimens: a pilot exploratory study

**DOI:** 10.1186/s12866-026-05386-w

**Published:** 2026-07-07

**Authors:** Nazym Syrym, Akbope Abdykalyk, Sabit Kokanov, Bolat Yespembetov, Aziz Nakhanov, Aibol Terebay, Alinur Toleukhan, Sabira Alpysbayeva, Aktoty Anarbekova, Makhpal Sarmykova, Yeldos Serikbay, Bekzat Yerzhigit, Kali Tileukhanov, Aigerim Zhakypbek, Azamat Abdimukhtar

**Affiliations:** Research Institute for Biological Safety Problems, National Holding QazBioPharm, Gvardeisky, 080409 Kazakhstan

**Keywords:** Syphilis, *Treponema pallidum*, Rabbit inoculation model, Molecular characterization, Real-time PCR, 16S rRNA, Phylogenetic analysis, Dark-field microscopy, Sanger sequencing, Experimental infection

## Abstract

**Background:**

Syphilis remains a major sexually transmitted infection worldwide. *Treponema pallidum* subspecies *pallidum* is difficult to investigate under conventional laboratory cultivation conditions, which limits experimental and molecular studies involving clinical material. This pilot exploratory study aimed to obtain preliminary molecular evidence of *Treponema pallidum*-related rabbit-derived material following inoculation with human chancre-derived clinical specimens. Chancre-derived clinical material was examined by dark-field microscopy and used for experimental intratesticular inoculation in rabbits, followed by real-time polymerase chain reaction (PCR), conventional PCR targeting partial 16 S ribosomal RNA gene fragments, Sanger sequencing, and phylogenetic analysis.

**Results:**

Thin spiral-shaped structures morphologically consistent with treponemal organisms were observed in selected clinical specimens by dark-field microscopy. Real-time PCR demonstrated amplification signals consistent with *Treponema pallidum*-related DNA in three rabbit-derived samples following initial inoculation. Because inflammatory findings remained limited, these PCR-positive rabbits subsequently received secondary intratesticular inoculation using cryopreserved chancre-derived material as an exploratory modification intended to improve recovery of rabbit-derived material for downstream molecular analysis. Following secondary inoculation, these PCR-positive rabbits developed unilateral orchitis-like inflammatory changes involving the secondarily inoculated (contralateral) testis. Conventional PCR generated partial 16 S rRNA gene amplicons of the expected size (~ 1.5 kb) in PCR-positive samples. Sequencing analysis demonstrated high similarity to published *Treponema pallidum* reference sequences, with sequence identity ranging from approximately 99.45% to 100%. Phylogenetic analysis demonstrated clustering of the obtained sequences with published *Treponema pallidum* reference sequences retrieved from GenBank.

**Conclusions:**

The combined use of dark-field microscopy, experimental rabbit inoculation, species-specific real-time PCR, sequencing, and phylogenetic analysis enabled preliminary molecular detection and partial sequence-based characterization of rabbit-derived *Treponema pallidum*-related material obtained following inoculation with chancre-derived clinical specimens. Although exploratory and limited by the small number of positive samples and partial 16 S ribosomal RNA gene analysis, the findings provide preliminary molecular evidence supporting the potential usefulness of rabbit-based experimental approaches for downstream molecular investigations.

## Background

Syphilis remains one of the most important bacterial sexually transmitted infections worldwide and continues to represent a major public health concern despite the availability of effective antimicrobial therapy. According to the World Health Organization, millions of new syphilis cases are reported annually, and incidence rates have increased in several geographic regions in recent years [1]. Untreated infection may lead to severe complications, including neurosyphilis, cardiovascular manifestations, adverse pregnancy outcomes, and congenital infection [2, 3].

The causative agent of syphilis, *Treponema pallidum* subspecies *pallidum*, is a highly invasive spirochete capable of rapid dissemination and long-term persistence within host tissues [4]. The organism has a relatively small genome and limited metabolic capacity, making it highly dependent on the host environment for survival [5]. These biological features contribute to the technical difficulties associated with laboratory investigation of *T. pallidum*.

Direct detection of *T. pallidum* in clinical specimens remains challenging. Dark-field microscopy has historically been used for visualization of treponemal organisms in chancre-derived material during primary syphilis [6]. However, the sensitivity of this method depends heavily on specimen quality, rapid processing, bacterial burden, and operator experience [3]. In addition, dark-field microscopy alone cannot reliably distinguish pathogenic *T. pallidum* from morphologically similar nonpathogenic treponemes.

Because of these limitations, molecular diagnostic methods have become increasingly important in syphilis research and laboratory diagnostics. PCR-based assays targeting *T. pallidum*-specific genomic regions have demonstrated improved sensitivity and specificity, particularly in lesion-derived specimens collected during early stages of infection [7, 8]. Real-time PCR is now widely used for molecular confirmation of *T. pallidum* DNA in clinical material [8].

Sequencing and phylogenetic analysis also play an important role in characterization of *T. pallidum*-related material and investigation of molecular epidemiology. Molecular typing studies have demonstrated geographic variability among circulating strains and contributed to improved understanding of transmission dynamics and strain diversity [9, 10]. More recently, whole-genome sequencing approaches have provided additional opportunities for studying the genetic diversity and evolutionary relationships of *T. pallidum* populations [11, 12]. It should be noted, however, that the partial 16 S rRNA gene is highly conserved among spirochetes and cannot reliably distinguish *T. pallidum* from closely related treponemes or resolve differences at the strain level; sequence-based findings derived from this marker are therefore best interpreted as sequences showing high similarity to published.

Despite advances in molecular diagnostics, rabbit models continue to be used in experimental studies involving *T. pallidum*. Rabbit passage has historically been applied for experimental recovery and amplification of treponemal material obtained from clinical specimens because the organism remains difficult to maintain under conventional bacteriological cultivation conditions [13]. Experimental rabbit infection models have also contributed to understanding the dissemination and persistence of *T. pallidum* infection [14].

Long-term in vitro cultivation systems for *T. pallidum* have recently been described and represent an important advance in treponemal research [15]. However, these systems remain technically demanding and are not yet widely implemented in routine laboratory practice. As a result, rabbit-based experimental approaches continue to be used when additional treponemal material is required for downstream molecular analyses [16]. Direct molecular characterization of treponemal DNA from human lesion specimens, including targeted DNA enrichment and direct sequencing approaches, is increasingly applied but remains constrained by the low abundance of treponemal DNA and the predominance of host-derived genetic material in clinical material. Experimental rabbit inoculation was therefore selected in the present study as a complementary approach intended to recover and enrich treponemal material from chancre-derived specimens for subsequent molecular analysis, rather than as a substitute for direct molecular methods.

At the same time, molecular data regarding *T. pallidum*-related clinical material remain limited in several geographic regions, including Central Asia. Additional studies integrating experimental rabbit models with molecular methods may therefore provide useful information for characterization of treponemal material obtained from clinical specimens.

The present study was designed as a pilot exploratory experimental investigation aimed at obtaining preliminary molecular evidence of *Treponema pallidum*-related material in rabbit-derived samples following inoculation with human chancre-derived clinical specimens.

The primary objective of the study was to investigate whether *Treponema pallidum*-related DNA could be detected in rabbit-derived material after experimental inoculation with lesion-derived clinical specimens.

Secondary objectives included evaluation of the suitability of rabbit-derived material for downstream molecular analyses, including real-time PCR, partial 16 S rRNA gene sequencing, and phylogenetic characterization.

To address these objectives, we combined dark-field microscopy, experimental rabbit inoculation, molecular detection methods, sequencing, and phylogenetic analysis for characterization of rabbit-derived *Treponema pallidum*-related material.

## Methods

### Collection and transport of chancre-derived clinical material

Chancre-derived clinical specimens were obtained from human patients with clinically suspected syphilitic lesions after written informed consent. Patient-identifying information was not included in the study dataset, and all specimens were coded before laboratory analysis. Clinical diagnosis was established by qualified clinicians based on characteristic lesion findings and routine serological and molecular testing performed at the participating clinical center.

Before sample collection, the lesion surface was gently cleaned using sterile gauze moistened with sterile 0.9% sodium chloride solution in order to reduce superficial contamination. Material was collected from the base and active margins of the lesion using sterile swabs or gentle scraping with a sterile disposable instrument. In cases where sufficient exudate was present, lesion fluid was collected directly into sterile tubes.

Immediately after collection, specimens were transferred into sterile microtubes containing chilled sterile 0.9% sodium chloride solution and transported to the laboratory under cooled conditions (2–8 °C). Samples were processed within 2–4 h after collection in order to minimize degradation of treponemal DNA and cellular material. Following collection, a portion of each clinical specimen was processed immediately for microscopy and experimental inoculation, while the remaining material intended for subsequent experimental procedures was transferred into cryoprotective preservation medium and stored at -80 °C until use. Repeated freeze-thaw cycles were avoided.

A total of 10 clinical specimens were included in the study. All specimens were chancre-derived material obtained from human patients with clinically suspected syphilis, and the clinical and laboratory characteristics of each specimen are summarized in Table 1.

**Table 1:** Clinical and laboratory characteristics of chancre-derived specimens included in the study

Specimen ID	Syphilis stage	Lesion site	Routine serology*	Clinical real-time PCR**	Dark-field microscopy	Rabbit-derived real-time PCR***
TP-24-1	Primary	Penile chancre	Positive	Positive	++	+
TP-24-2	Primary	Penile lesion	Positive	Positive	+	−
TP-24-3	Primary	Genital ulcer	Positive	Positive	+++	−
TP-24-4	Secondary	Perianal lesion	Positive	Positive	++	−
TP-24-5	Primary	Multiple penile chancres	Positive	Positive	++	−
TP-24-6	Primary	Penile chancre	Positive	Positive	+	−
TP-24-7	Secondary	Perianal ulcer	Positive	Positive	+++	+
TP-24-8	Primary	Penile lesion	Positive	Positive	++	+
TP-24-9	Primary	Genital chancre	Positive	Positive	+	−
TP-24-10	Primary	Penile ulcer	Positive	Positive	++	−

*Routine serological testing was performed as part of clinical evaluation at the participating clinical center

**Clinical real-time PCR refers to molecular detection of Treponema pallidum DNA in chancre-derived clinical specimens prior to rabbit inoculation. Testing was performed as part of routine diagnostic evaluation at the participating clinical center. Original diagnostic Ct values were not available for standardized comparative analysis; therefore, clinical PCR results are reported qualitatively as positive

***Rabbit-derived real-time PCR refers to the interim screening result obtained from rabbit-derived testicular material collected 25 days after primary inoculation from the initially inoculated (left) testis. Ct values for the three positive samples (Table 2) were obtained separately at the terminal sampling point

### Dark-field microscopy

Fresh clinical specimens were examined immediately after collection using dark-field microscopy.

A drop of specimen material was placed onto a clean glass slide, covered with a coverslip, and examined under dark-field illumination using a Primo Star microscope (Carl Zeiss, Oberkochen, Germany) at ×400 magnification.

Thin spiral-shaped structures morphologically consistent with treponemal organisms were observed in selected specimens. Microscopic findings were recorded semi-quantitatively as low (+), moderate (++), or abundant (+++).

### Experimental animals

Adult male rabbits (*Oryctolagus cuniculus*) aged 3–4 months and weighing 2.5–3.0 kg were used in the experimental inoculation study. The animals were obtained from the institutional animal facility of the Research Institute for Biological Safety Problems, Republic of Kazakhstan. Animals were clinically healthy and housed individually under standard laboratory conditions at a temperature of 20–22 °C and relative humidity of 40–60% under a 12 h light/dark cycle. Standard laboratory feed and water were provided *ad libitum.*

Prior to experimental procedures, rabbits were acclimatized for at least 5 days. Animals were monitored daily throughout the study period for general clinical condition, behavior, food and water intake, and the development of local inflammatory changes following inoculation. Rabbits were observed for up to 8 weeks after inoculation. Animals demonstrating severe distress or unexpected clinical deterioration would have been humanely euthanized according to institutional ethical guidelines; however, no such cases occurred during the study. All procedures involving animals were performed under conditions intended to minimize discomfort and stress.

A total of 13 rabbits were included in the study, including 10 experimentally inoculated rabbits and 3 negative control rabbits.

### Study design and endpoints

The present study was conducted as a pilot exploratory experimental investigation combining rabbit inoculation with molecular characterization approaches for analysis of *Treponema pallidum*-related material obtained from chancre-derived clinical specimens.

The primary endpoint of the study was detection of *Treponema pallidum*-related DNA in rabbit-derived samples by real-time PCR following experimental inoculation.

Secondary endpoints included visualization of spiral-shaped structures morphologically consistent with treponemal organisms by dark-field microscopy, development of local inflammatory changes after inoculation, generation of partial 16 S rRNA gene amplicons, sequence similarity with published *Treponema pallidum* reference sequences and phylogenetic clustering of the obtained sequences with reference *Treponema pallidum* strains retrieved from GenBank. The study included 10 experimentally inoculated rabbits and 3 negative control rabbits inoculated with sterile 0.9% sodium chloride solution.

### Preparation of inoculum

Clinical material obtained from chancre lesions was used as the source material for experimental inoculation.

Specimens were suspended in 0.5-1.0 mL of sterile 0.9% sodium chloride solution and gently mixed to obtain a homogeneous suspension. Large tissue fragments and debris were removed by low-speed centrifugation at 1,000 × g for 5 min. The resulting supernatant was used immediately for inoculation.

### Rabbit inoculation

Rabbits were initially inoculated intratesticularly in the left testis using 0.5 mL of the prepared suspension administered aseptically with sterile disposable syringes. To minimize pain and distress, the procedure was performed under general anaesthesia induced by intramuscular administration of ketamine hydrochloride (35 mg/kg) and xylazine hydrochloride (5 mg/kg). The depth of anaesthesia was assessed before inoculation by monitoring the pedal withdrawal reflex, muscle tone, respiratory pattern and response to handling. Negative control rabbits received sterile 0.9% sodium chloride solution by the same route, volume, and experimental conditions. Animals were monitored during the procedure until full recovery from anaesthesia, and daily thereafter for local inflammatory changes including enlargement, induration and asymmetry of the inoculated testis.

Following primary intratesticular inoculation into the left testis, rabbits were monitored daily for local inflammatory changes. For interim molecular screening, rabbit-derived testicular material was collected aseptically from the initially inoculated testicular site 25 days after primary inoculation and analysed by real-time PCR. This interim sampling was performed as a non-terminal procedure under general anaesthesia induced by intramuscular administration of ketamine hydrochloride (35 mg/kg) and xylazine hydrochloride (5 mg/kg). After disinfection of the scrotal skin, material was obtained from the primarily inoculated testis by fine-needle aspiration using a sterile needle and syringe, and the aspirate was processed immediately for molecular analysis. Rabbits were monitored during recovery from anaesthesia and subsequently observed for local bleeding, swelling, appetite, activity, and general condition. Three rabbits showed positive real-time PCR findings despite limited local inflammatory changes. These PCR-positive rabbits subsequently received secondary intratesticular inoculation into the right, contralateral testis using cryopreserved chancre-derived material from the corresponding human clinical specimen. The contralateral testis was selected to avoid repeated manipulation of the initially inoculated site and to evaluate a newly inoculated local site under the same experimental conditions. This exploratory secondary procedure was performed to increase the likelihood of obtaining sufficient rabbit-derived material for downstream molecular analysis and was not intended to demonstrate sustained in vivo propagation or strain isolation. Terminal tissue collection was performed 17 days after secondary inoculation. At the terminal stage, rabbits were humanely euthanised before tissue collection in accordance with the approved institutional animal ethics protocol. Before euthanasia, animals were placed under deep general anaesthesia using ketamine hydrochloride (35 mg/kg) and xylazine hydrochloride (5 mg/kg), administered intramuscularly. Adequate anaesthetic depth was confirmed by the absence of the pedal withdrawal reflex, corneal reflex, and response to handling. Euthanasia was performed by intravenous overdose of pentobarbital sodium (60 mg/kg) under deep anaesthesia. Death was confirmed by the absence of respiration and heartbeat. Testicular tissue was collected aseptically only after death had been confirmed and was processed for subsequent molecular analyses. The remaining seven PCR-negative inoculated rabbits and the three negative control rabbits were humanely euthanised at the end of the planned observation period using the same anaesthesia and euthanasia procedure, and their testicular tissue was collected and processed identically for molecular analysis. The Ct values reported in Table 2 refer to rabbit-derived samples collected at the terminal sampling point, 17 days after secondary inoculation, from the contralateral testis that received the secondary inoculation and showed orchitis-like inflammatory changes. Interim screening samples collected on day 25 after primary inoculation were obtained from the primarily inoculated testis by fine-needle aspiration and are reported separately.

### Preparation of rabbit-derived material for molecular analysis

Testicular tissue collected from rabbits with local inflammatory changes was processed under aseptic conditions for molecular analysis. Rabbit-derived terminal testicular tissue was used for conventional PCR, sequencing, and phylogenetic analysis.

Small tissue fragments were suspended in sterile 0.9% sodium chloride solution and gently disrupted to obtain a tissue suspension. Coarse tissue debris was removed by low-speed centrifugation at 800 × g for 10 min, and the resulting supernatant was used for DNA extraction and subsequent molecular analyses.

### DNA extraction

Genomic DNA was extracted from both clinical specimens and rabbit-derived samples using the GeneJET Genomic DNA Purification Kit (Thermo Fisher Scientific, Waltham, MA, USA) according to the manufacturer’s instructions.

Briefly, 200 µL of sample was subjected to lysis followed by column-based purification. DNA was eluted in 50 µL of elution buffer.

DNA concentration and purity were assessed using a Nabi UV/Vis Nano Spectrophotometer (MicroDigital Co., Ltd., Seongnam, South Korea).

Negative extraction controls were included throughout the extraction procedure.

### Real-time PCR detection

Detection of *Treponema pallidum* DNA was performed using the GeneProof *Treponema pallidum* PCR Kit (GeneProof, Brno, Czech Republic) according to the manufacturer’s instructions.The assay targets the *polA* gene (encoding DNA polymerase I) of *Treponema pallidum*, with target detection in the FAM channel and a co-amplified internal control monitoring DNA extraction efficiency and PCR inhibition in the HEX/JOE/VIC channel.

PCR reactions were prepared in a total volume of 40 µL, comprising 30 µL of Master Mix and 10 µL of extracted DNA template (or positive control), in accordance with the manufacturer’s instructions. Amplification was performed on an Applied Biosystems QuantStudio 5, real-time PCR system (Thermo Fisher Scientific, Waltham, MA, USA) using the kit DNA PCR profile: uracil-N-glycosylase decontamination at 37 °C for 2 min; initial denaturation at 95 °C for 10 min; and 45 cycles of denaturation at 95 °C for 5 s, annealing at 60 °C for 40 s, and extension at 72 °C for 20 s, with fluorescence acquisition in the FAM (target) and internal-control channels.

Because the assay is a qualitative test, the manufacturer does not define a numerical Ct cutoff; a sample was interpreted as positive when a characteristic exponential amplification curve was detected in the FAM *(Treponema pallidum)* channel within the 45-cycle profile, provided that the internal control amplified validly and the positive and negative run controls gave the expected results. Each run included positive, negative, and no-template controls.

### Conventional PCR and gel electrophoresis

PCR amplification of partial 16 S rRNA gene fragments was performed for subsequent sequencing and molecular characterization.

Universal bacterial primers targeting the 16 S rRNA gene were used for amplification prior to sequencing as previously described by Lane [17]: forward primer 27 F (5′-AGAGTTTGATCMTGGCTCAG-3′) and reverse primer 1492R (5′-TACGGYTACCTTGTTACGACTT-3′).

PCR reactions were carried out in a total volume of 25 µL containing 1× PCR buffer, 2.0 mM MgCl₂, 0.2 mM of each dNTP, 0.4 µM of each primer (27 F and 1492R), 1.25 U of Taq DNA polymerase (Thermo Fisher Scientific, Waltham, MA, USA), and approximately 50 ng of template DNA.

Thermal cycling conditions included initial denaturation at 95 °C for 5 min, followed by 35 cycles of denaturation at 95 °C for 30 s, annealing at 55 °C for 30 s, and extension at 72 °C for 1 min, with a final extension at 72 °C for 5 min.

PCR products were analyzed by electrophoresis in a 1.5% agarose gel prepared using UltraPure Agarose (Invitrogen, Carlsbad, CA, USA) and visualized under UV illumination. Amplicons of the expected size were selected for sequencing.

### Sequencing and phylogenetic analysis

PCR products corresponding to the expected amplicon size were purified prior to sequencing and subjected to Sanger sequencing using the BigDye Terminator v3.1 Cycle Sequencing Kit (Applied Biosystems, Foster City, CA, USA).

Sequence chromatograms were manually inspected prior to analysis. Sequences were analyzed using the BLAST algorithm against the NCBI nucleotide database.

Consensus sequences were generated and used for comparative sequence and phylogenetic analyses.

Phylogenetic analysis was performed using MEGA software version 11. Phylogenetic trees were constructed using the Neighbor-Joining method with 1000 bootstrap replicates based on the Kimura 2-parameter model.

### Contamination control

Pre-PCR and post-PCR procedures were performed in physically separated laboratory areas. Dedicated equipment and aerosol-resistant filter tips were used throughout the study.

Negative extraction controls and no-template PCR controls were included at all stages of amplification and sequencing procedures.

### Statistical analysis

Because of the pilot exploratory nature of the study and the limited number of positive samples, data were analyzed descriptively.

No formal sample size or power calculation was performed. The number of clinical specimens and experimental animals was determined by feasibility, specimen availability, and biosafety considerations.

A schematic overview of the experimental workflow used in the present study is presented in Fig. 1.

**Fig. 1:**
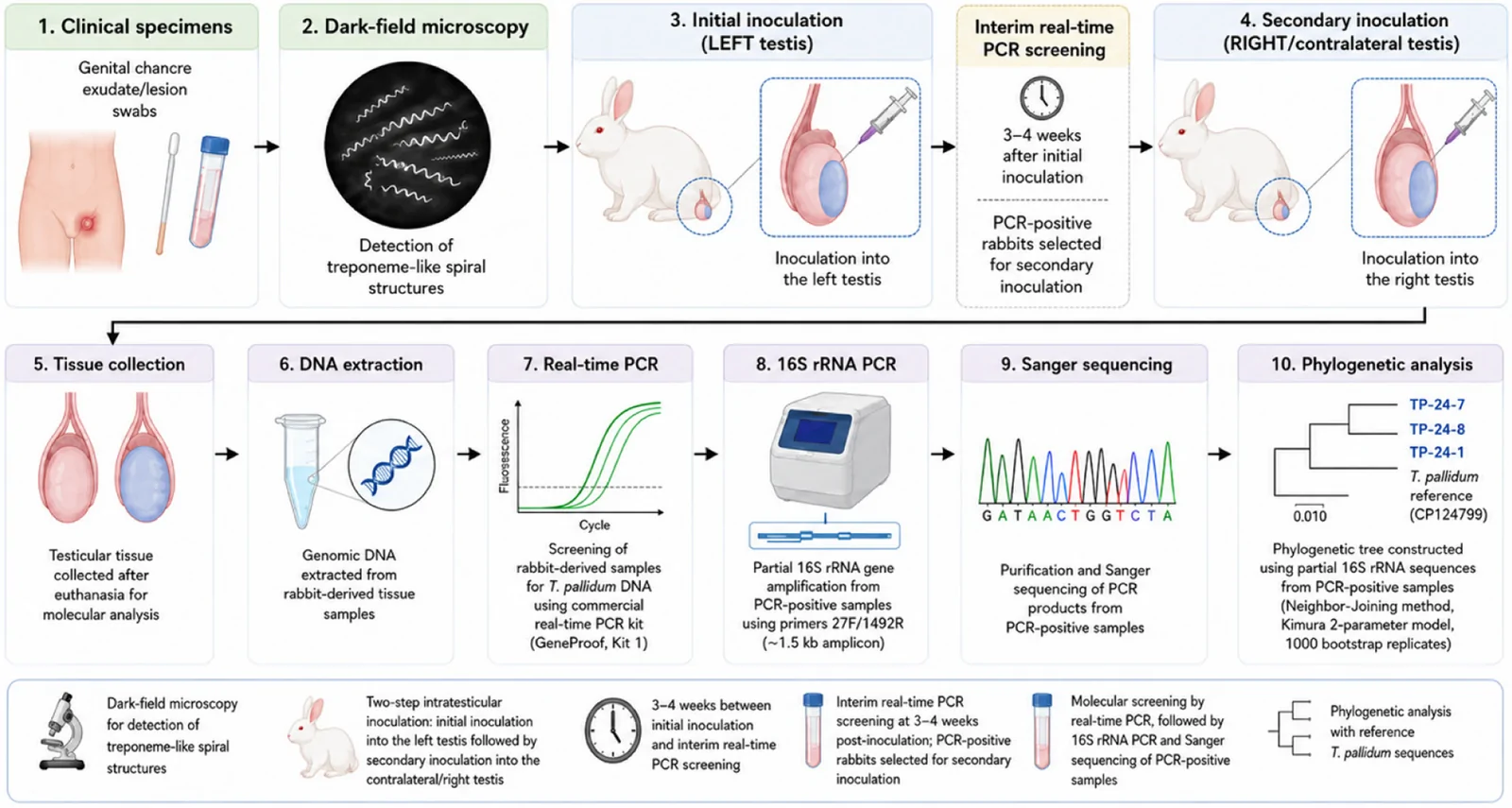
Experimental workflow for molecular characterization of rabbit-derived *Treponema pallidum*-related material. Chancre-derived clinical specimens were examined by dark-field microscopy and used for initial intratesticular inoculation into the left testis. Rabbit-derived samples were analysed by real-time PCR, and PCR-positive rabbits subsequently received secondary intratesticular inoculation in the contralateral testis using preserved chancre-derived material from the corresponding clinical specimen. At the end of the observation period, testicular tissue was collected after humane euthanasia and processed for molecular analysis. PCR-positive rabbit-derived samples were used for partial 16 S rRNA PCR, Sanger sequencing, and phylogenetic analysis

## Results

### Dark-field microscopy of chancre-derived clinical material

Fresh chancre-derived clinical specimens were examined by dark-field microscopy immediately after collection.

Thin spiral-shaped structures morphologically consistent with treponemal organisms were observed in several clinical specimens (Fig. 2). The abundance of observed structures varied among samples and was recorded semi-quantitatively from low (+) to abundant (+++) levels. In some specimens, only small numbers of spiral-shaped structures were identified, whereas others showed more abundant microscopic findings.

**Fig. 2:**
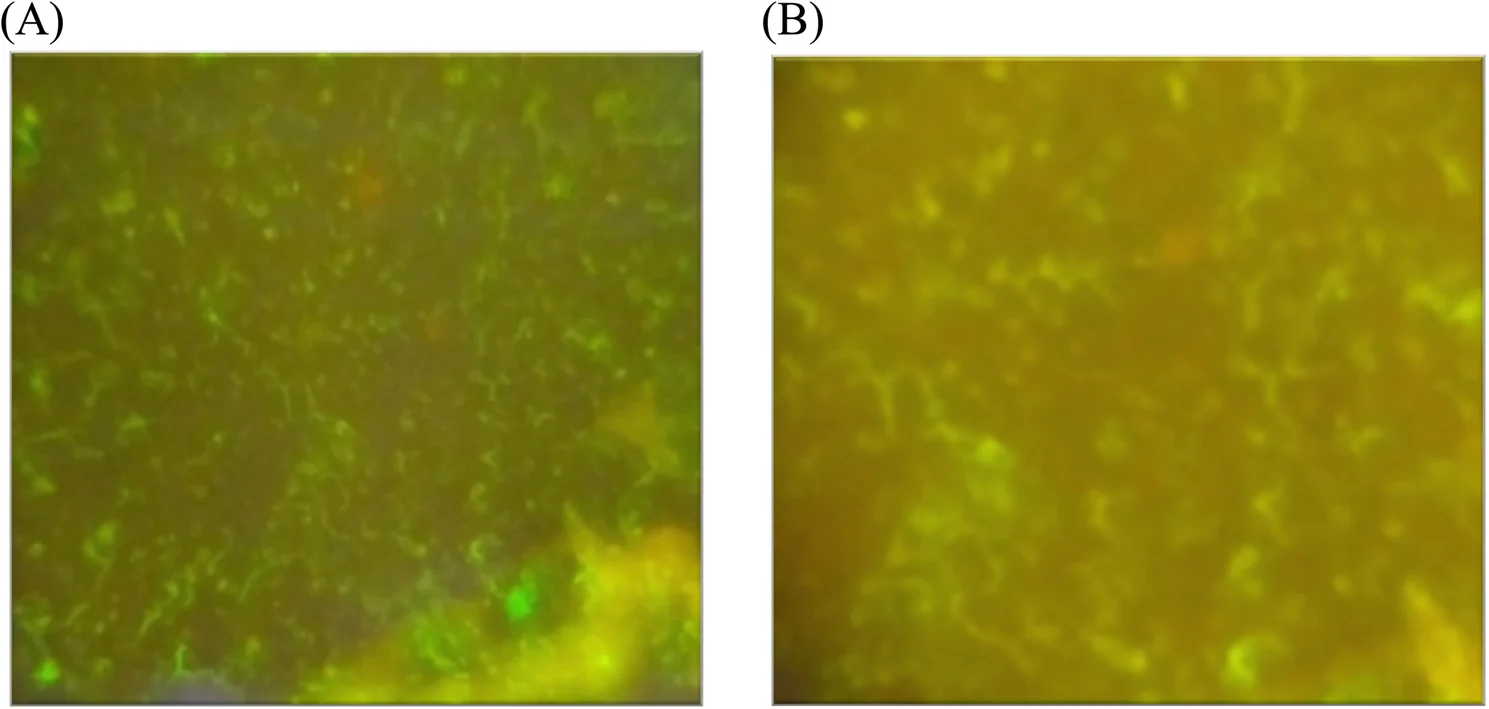
Representative dark-field microscopy images obtained from chancre-derived clinical specimens. Thin spiral-shaped structures morphologically consistent with treponemal organisms were observed in selected specimens. Panels **A** and **B** show representative microscopic fields from different clinical samples

### Rabbit inoculation and clinical observations

A total of 10 rabbits were inoculated intratesticularly with chancre-derived clinical material and monitored daily throughout the study period. Clinical and laboratory characteristics of the chancre-derived specimens used for rabbit inoculation are summarized in Table 1. All chancre-derived specimens included in the study had been previously characterized as *Treponema pallidum*-positive during routine clinical molecular evaluation performed at the participating clinical center. 

Dark-field microscopy findings were recorded semi-quantitatively as low (+), moderate (++), or abundant (+++) detection of spiral-shaped structures morphologically consistent with treponemal organisms.

Following primary intratesticular inoculation into the left testis, no pronounced inflammatory changes were observed during the first several weeks. Interim real-time PCR screening was performed on rabbit-derived testicular material collected 25 days after primary inoculation. *Treponema pallidum*-related DNA was detected in three of ten experimentally inoculated rabbits. Because local inflammatory findings remained limited, these three PCR-positive rabbits subsequently received secondary intratesticular inoculation into the right, contralateral testis using cryopreserved chancre-derived clinical material from the corresponding human specimen. This secondary procedure was interpreted as an exploratory methodological modification and was not considered evidence of sustained in vivo propagation. Following secondary inoculation, unilateral orchitis-like inflammatory changes became apparent within approximately 1–2 weeks and involved the right, secondarily inoculated contralateral testis. These changes included enlargement, induration, and asymmetry of the affected testis. Terminal testicular tissue was collected 17 days after secondary inoculation for downstream molecular analysis. Negative control rabbits inoculated with sterile 0.9% sodium chloride solution demonstrated no local inflammatory changes and no detectable *Treponema pallidum*-related DNA amplification by real-time PCR. No severe systemic abnormalities or unexpected mortality were observed during the study period.

### Real-time PCR detection of *Treponema pallidum*.

Real-time PCR analysis was performed on rabbit-derived samples obtained from experimentally inoculated rabbits. Amplification signals consistent with *Treponema pallidum*-related DNA were detected in three rabbit-derived samples following inoculation with chancre-derived clinical material.

Among the analyzed rabbit-derived specimens, three demonstrated amplification profiles interpreted as positive according to the manufacturer’s recommended interpretation criteria.

DNA extracted from the PCR-positive rabbit-derived terminal testicular tissue samples showed concentrations ranging from 70.3 to 83.7 ng/µL. Positive rabbit-derived samples demonstrated Ct values ranging from approximately 19 to 32 cycles, consistent with variability in detectable target DNA among specimens (Table 2; Fig. 3). These findings represented the primary molecular endpoint of the present exploratory study. A direct correlation between the clinical dark-field microscopy score and the rabbit-derived Ct value was not expected, because the dark-field score refers to the original human chancre-derived specimen prior to inoculation, whereas the Ct value reflects treponemal DNA recovered from rabbit-derived testicular tissue after inoculation, processing, and DNA extraction.

**Table 2:** Molecular characteristics of PCR-positive rabbit-derived samples

Sample ID	Clinical dark-field microscopy score*	Rabbit-derived real-time PCR result	Rabbit-derived real-time PCR Ct	DNA concentration (ng/µL)**
TP-24-1	++	Positive	19.13	70.3
TP-24-7	+++	Positive	28.60	83.7
TP-24-8	++	Positive	32.30	78.5

Ct values were obtained from rabbit-derived terminal testicular tissue collected from the secondarily inoculated (right) testis. The assay positive control included in the same run showed a Ct of 17.02

*Dark-field microscopy scores refer to chancre-derived clinical specimens before rabbit inoculation

**DNA concentration refers to DNA extracted from PCR-positive terminal testicular tissue samples. Clinical diagnostic Ct values were not available for standardized comparative analysis

Negative extraction controls and no-template controls demonstrated no detectable amplification throughout the analysis, supporting the absence of detectable contamination during DNA extraction and amplification procedures.

Samples demonstrating positive amplification were subsequently selected for conventional PCR amplification of partial 16 S rRNA gene fragments followed by sequencing and phylogenetic analysis.

**Fig. 3:**
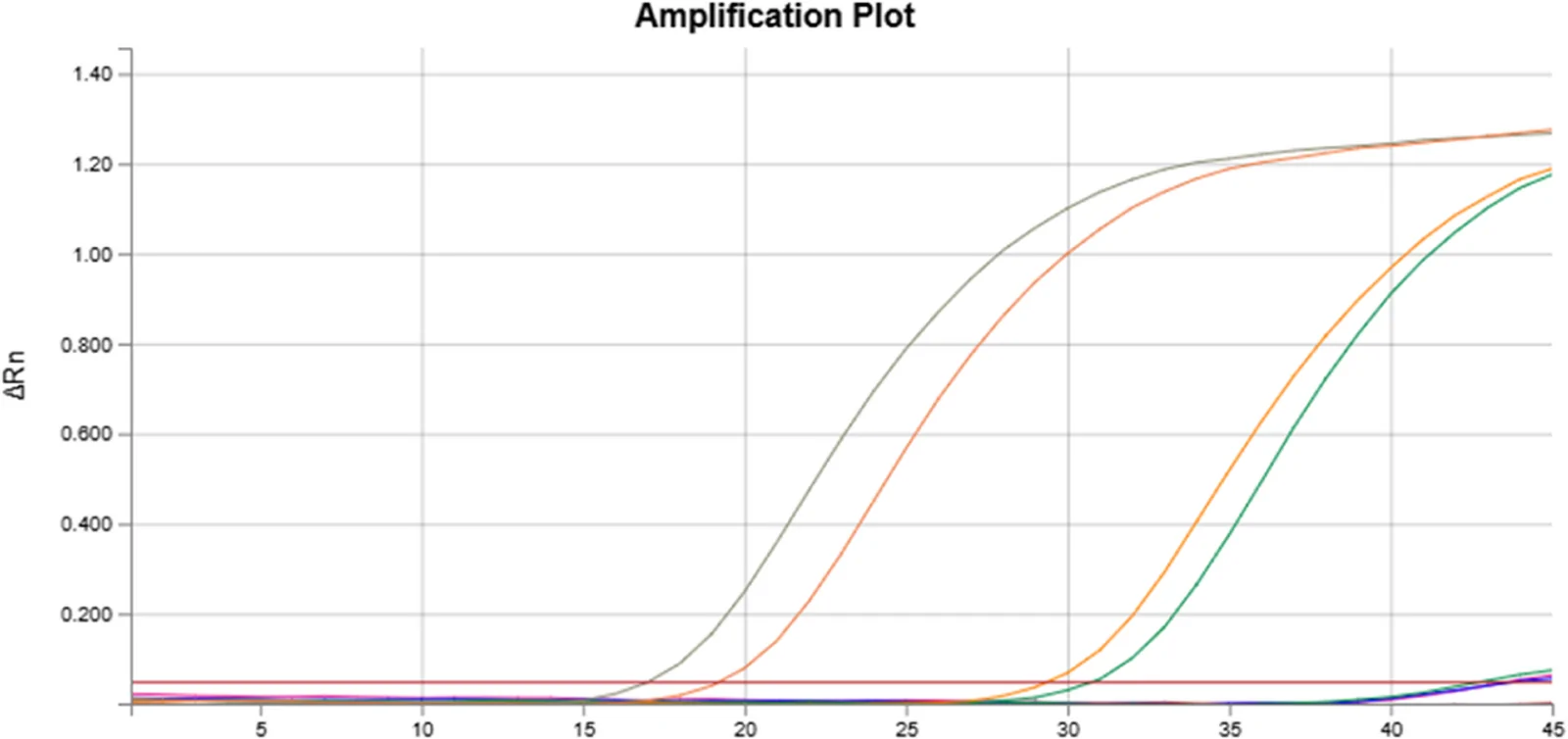
Representative real-time PCR amplification curves obtained from rabbit-derived samples following inoculation with chancre-derived clinical material. Distinct amplification profiles consistent with positive *Treponema pallidum*-specific real-time PCR findings were observed in selected rabbit-derived samples, whereas negative extraction controls and no-template controls demonstrated no detectable amplification throughout the analysis

### Conventional PCR and electrophoretic analysis

PCR amplification of partial 16 S rRNA gene fragments generated products of the expected size (~ 1.5 kb) in samples previously demonstrating positive real-time PCR results. Distinct amplification products corresponding to the expected amplicon size were obtained from selected rabbit-derived samples and used for subsequent sequencing analysis. No visible amplification products were observed in negative controls.

### Sequencing and molecular characterization

PCR products obtained from selected rabbit-derived samples were subjected to Sanger sequencing followed by comparative sequence analysis using the BLAST algorithm against the NCBI nucleotide database.

BLAST analysis demonstrated high similarity between the obtained partial 16 S rRNA gene sequences and published *Treponema pallidum* reference sequences available in GenBank (Table 3). The analyzed sequences showed 100% query coverage and sequence identity ranging from approximately 99.45% to 100%.

**Table 3:** Sequence similarity analysis of rabbit-derived samples based on partial 16 S rRNA gene sequencing and BLAST comparison with GenBank reference sequences

Sample ID	Closest matching reference sequence	Accession number	Query coverage (%)	Identity (%)
TP-24-1	*Treponema pallidum*	CP073566.2	100	100
TP-24-1	*Treponema pallidum*	CP094485.1	100	99.70
TP-24-8	*Treponema pallidum*	CP094485.1	100	99.87
TP-24-8	*Treponema pallidum*	CP156971.1	100	99.45
TP-24-7	*Treponema pallidum*	CP094485.1	100	99.98
TP-24-7	*Treponema pallidum*	CP073553.2	100	99.74

*Only the highest-scoring representative BLAST matches for each analyzed sample are shown

### Phylogenetic analysis

Phylogenetic analysis based on partial 16 S rRNA gene sequences demonstrated clustering of the obtained sequences with reference *Treponema pallidum* sequences retrieved from GenBank.

The analyzed sequences clustered closely with published *Treponema pallidum* reference strains within the phylogenetic tree. Although the analyzed 16 S rRNA fragment demonstrated a high degree of sequence conservation, the phylogenetic findings supported the molecular similarity of the analyzed rabbit-derived samples to *Treponema pallidum (*Fig. 4*).*

**Fig. 4:**
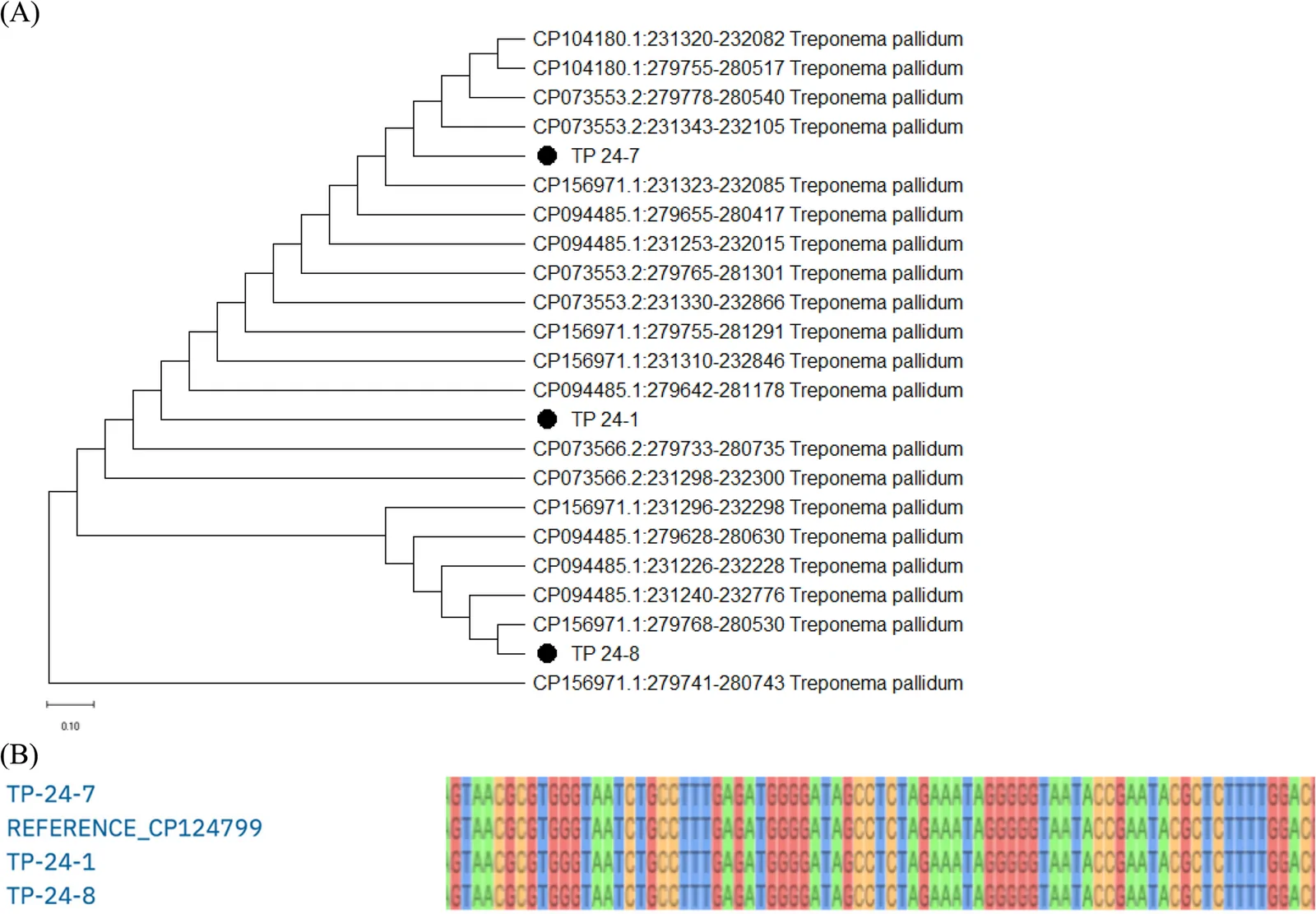
Molecular characterization of rabbit-derived *Treponema pallidum*-related sequences. **A** Phylogenetic tree constructed using partial 16 S rRNA gene sequences obtained from rabbit-derived samples and reference Treponema pallidum sequences retrieved from GenBank. Phylogenetic analysis was performed using the Neighbor-Joining method with 1000 bootstrap replicates based on the Kimura 2-parameter model. **B** Representative multiple sequence alignment of partial 16 S rRNA gene sequences obtained from rabbit-derived samples and the reference Treponema pallidum sequence CP124799 retrieved from GenBank. The displayed alignment demonstrates a highly conserved nucleotide region shared between rabbit-derived sequences and the reference sequence

## Discussion

The present pilot study combined experimental rabbit inoculation with molecular approaches to characterize rabbit-derived material obtained following inoculation with chancre-derived clinical specimens.

In the present study, dark-field microscopy findings were considered supportive preliminary observations rather than definitive evidence of *Treponema pallidum.* Real-time PCR served as the primary molecular detection method, whereas sequencing and phylogenetic analyses were used as supplementary approaches for characterization of rabbit-derived material. The combined use of complementary methods was considered important because direct investigation of *T. pallidum* remains technically challenging due to the organism’s limited ability to survive under conventional bacteriological cultivation conditions [3, 13].

As outlined above, dark-field microscopy is a classical but strongly operator- and specimen-dependent method that cannot, by itself, distinguish pathogenic *T. pallidum* from morphologically similar nonpathogenic treponemes; current clinical guidance and recent updates therefore support interpreting microscopic findings together with molecular or serological methods rather than as a single confirmatory approach [3, 4]. Accordingly, the spiral-shaped structures observed here were interpreted conservatively as structures morphologically consistent with treponemal organisms. Dark-field microscopy was performed only on fresh chancre-derived clinical specimens before rabbit inoculation. It was not performed on rabbit-derived material (including both the day-25 aspirate and terminal testicular tissue) because the available rabbit-derived material was prioritized for DNA extraction and downstream molecular analyses, and tissue-derived suspensions containing host cells and debris are less suitable for reliable dark-field interpretation than fresh lesion exudate. Therefore, the present study did not assess the presence or motility of treponemal organisms in rabbit-derived material, and this should be considered an important limitation.

In the present study, the rabbit inoculation model was applied specifically to recover and enrich treponemal material from chancre-derived specimens for downstream molecular analysis, consistent with the continued use of rabbit-based approaches when *T. pallidum* cannot be readily maintained using conventional bacteriological cultivation methods [13, 14]. The observation that only a subset of inoculated animals yielded detectable treponemal DNA underscores that, although long-term in vitro cultivation systems have now been described [15], rabbit passage remains an imperfect but practically useful means of obtaining treponemal material for molecular investigations [16] rather than a reliable propagation system in its own right.

No pronounced local inflammatory changes were observed during the first several weeks following the initial inoculation procedure. More evident unilateral orchitis-like inflammatory changes involving the right, secondarily inoculated contralateral testis became apparent following secondary inoculation in PCR-positive rabbits. The severity and progression of inflammatory findings varied among rabbits during the observation period. Similar variability in rabbit responses following inoculation with treponemal material, including delayed inflammatory manifestations and variable molecular findings, has been described previously in experimental rabbit studies involving *T. pallidum* [14, 16, 18, 19]. Histopathological characterization of inflammatory lesions was not performed because this was beyond the scope of the present pilot molecular investigation; therefore, the observed orchitis-like changes were evaluated at the gross clinical level only. Importantly, no severe systemic abnormalities or unexpected mortality were observed during the study period.

Real-time PCR provided the primary molecular evidence supporting the presence of *Treponema pallidum-*related DNA in three PCR-positive rabbit-derived samples. Three samples demonstrated amplification profiles interpreted as positive according to the manufacturer’s criteria. Because the commercial assay provides qualitative results, positivity was defined by detection of a characteristic amplification curve in the *Treponema pallidum* (FAM) channel together with a valid internal control rather than by a fixed Ct threshold; negative extraction controls and no-template controls remained negative throughout the analysis. DNA extracted from PCR-positive rabbit-derived terminal testicular tissue samples showed concentrations ranging from 70.3 to 83.7 ng/µL, supporting the suitability of these extracts for downstream conventional PCR and sequencing analyses. Importantly, species-specific molecular detection relied primarily on the commercial *Treponema pallidum-*specific real-time PCR assay, whereas partial 16 S rRNA sequencing was used as a supplementary molecular characterization approach.

Sequencing analysis demonstrated high similarity between the obtained partial 16 S rRNA gene fragments and published *T. pallidum* reference sequences available in GenBank. The analyzed sequences demonstrated approximately 99.45–100% similarity depending on the analyzed fragment and reference sequence. However, these findings should be interpreted cautiously because the 16 S rRNA gene is highly conserved among closely related spirochetes and provides limited discriminatory power for definitive species-level or strain-level identification. Therefore, the sequencing findings presented here should not be interpreted as definitive evidence of sustained *Treponema pallidum* propagation in rabbits. Instead, partial 16 S rRNA sequencing was used as supportive molecular evidence complementing the real-time PCR findings, which were obtained with a commercial assay targeting the polA gene, and the overall experimental context. Future studies incorporating sequence-based analysis of additional *T. pallidum*-specific molecular targets, including *tpp47*,* polA*, or *tp0548*, may provide substantially higher-resolution molecular characterization.

Phylogenetic analysis demonstrated clustering of the obtained sequences with published *T. pallidum* reference sequences retrieved from GenBank. Although the analyzed sequences clustered closely with reference sequences, the conserved nature of the analyzed genomic region limits detailed strain-level interpretation. Additional molecular targets, multilocus sequence analysis, and whole-genome sequencing would provide substantially more detailed characterization of rabbit-derived treponemal material [9–12].

Several limitations of the present study should be acknowledged. The number of positive rabbit-derived samples was limited, and molecular characterization was based primarily on partial 16 S rRNA gene analysis. The study did not include multilocus sequence typing, whole-genome sequencing, quantitative bacterial load assessment, histopathological analysis, organism viability assessment, infectivity testing, or long-term serial passage experiments. Most importantly, because secondary inoculation was performed in selected PCR-positive rabbits using cryopreserved chancre-derived material from the corresponding human clinical specimen, the present study cannot distinguish between persistence of initially introduced DNA, DNA newly introduced during secondary inoculation, and true in vivo multiplication. Therefore, the present findings should be interpreted conservatively as molecular detection and characterization of *Treponema pallidum*-related rabbit-derived material following experimental inoculation, rather than direct evidence of sustained in vivo propagation or successful strain isolation. The limited number of PCR-positive rabbit-derived samples may additionally reflect variability in treponemal burden among clinical specimens, differences in inoculum quality, specimen handling and preservation, or the known technical challenges associated with experimental investigation of *T. pallidum.*

Rabbit-derived samples demonstrating positive molecular findings represented only a subset of experimentally inoculated animals. The present study was designed primarily as an exploratory molecular investigation rather than a definitive propagation or infectivity study. Accordingly, the findings should be interpreted as preliminary molecular characterization of rabbit-derived material demonstrating molecular similarity to *Treponema pallidum*, rather than confirmation of sustained propagation capacity or establishment of a continuous experimental infection system.

Despite these limitations, the present study demonstrates the usefulness of combining rabbit-based experimental approaches with molecular methods, including real-time PCR, sequencing, and phylogenetic analysis, for characterization of rabbit-derived material obtained from clinical specimens. Because direct whole-genome characterization of *T. pallidum* from clinical material remains technically challenging due to the low abundance of treponemal DNA and the presence of host-derived genetic material, rabbit-based experimental approaches may provide additional opportunities for obtaining treponemal DNA suitable for downstream molecular investigations.

Given the scarcity of regional molecular data noted earlier, including for Central Asia, future studies involving larger numbers of clinical specimens, expanded molecular targets, multilocus sequence typing and whole-genome sequencing may contribute to improved understanding of the molecular epidemiology and genetic diversity of circulating *Treponema pallidum*-related strains in the region. Future studies should also incorporate additional *T. pallidum*-specific molecular targets, including *tpp47*,* polA*, and *tp0548*, together with absolute quantitative PCR using standard curves for copy number determination. This would allow treponemal burden to be assessed more directly and may help distinguish low-level residual DNA detection from increasing bacterial load consistent with in vivo multiplication. Where sufficient DNA quantity and quality are available, targeted DNA enrichment or whole-genome sequencing should also be considered for higher-resolution molecular characterization. The present findings should therefore be interpreted within the context of a preliminary exploratory molecular investigation rather than a definitive experimental propagation study.

## Conclusions

In the present study, combined microscopic, experimental, and molecular approaches were applied to analyze rabbit-derived material obtained following inoculation with human chancre-derived clinical specimens. The integration of dark-field microscopy, experimental rabbit inoculation, species-specific real-time PCR, sequencing, and phylogenetic analysis enabled preliminary molecular detection and partial sequence-based characterization of selected rabbit-derived samples demonstrating high similarity to published *Treponema pallidum* reference sequences.

Although the present study was exploratory and limited by the small number of positive samples, partial 16 S rRNA gene analysis, absence of rabbit-derived dark-field microscopy, absence of histopathological characterization, and lack of viability or long-term propagation assessment, the findings provide preliminary molecular evidence supporting the potential usefulness of rabbit-based experimental approaches for obtaining treponemal DNA suitable for downstream molecular investigations. Because secondary inoculation was performed in selected PCR-positive rabbits, these findings should not be interpreted as definitive evidence of sustained in vivo propagation, successful strain isolation, or strain-level confirmation.

Future studies incorporating expanded *T. pallidum*-specific molecular targets, including *tpp47*, *polA*, and *tp0548*, together with absolute quantitative PCR using standard curves, histopathological analysis, and whole-genome or targeted-enrichment sequencing may help provide a more comprehensive understanding of experimentally derived treponemal material and its molecular characteristics.

## Data Availability

The partial 16S rRNA gene sequences generated in this study have been deposited in GenBank under accession numbers PZ461046 (TP-24-1), PZ461047 (TP-24-8) and PZ461048 (TP-24-7). The remaining datasets generated and/or analysed during the current study are available from the corresponding author on reasonable request.
